# *In vitro* assessment of a novel additive manufactured titanium implant abutment

**DOI:** 10.4317/jced.57389

**Published:** 2021-02-01

**Authors:** Les Kalman

**Affiliations:** 1 Assistant Professor, Restorative Dentistry, Chair, Dental Outreach, Schulich School of Medicine & Dentistry, Western University, 1151 Richmond Street, London, Ontario, Canada

## Abstract

**Background:**

Dental implant temporization remains a clinical challenge. A novel abutment simplifying the procedure was developed, but conventional fabrication was costly and unpredictable. A workflow was developed to fabricate the novel abutment using additive manufacturing. This in vitro investigation assessed the additive manufactured (AM) novel abutments to conventionally manufactured (CM) abutments.

**Material and Methods:**

The AM abutments were fabricated in dental-grade titanium (Ti-6Al-4V) using Selective Laser Melting and were post-processed. The CM abutments were milled and subsequently laser welded manually. Pin strength of the abutments was assessed using a universal loading machine. Torque was measured by tightening the AM and CM abutments into dental implants within artificial bone.

**Results:**

Average pin strength was 364.4 N for the AM abutments and 62.5 N for the CM abutments. Average torque was 49.9 Ncm for the AM abutments and 62.9 Ncm for the CM abutments. AM abutment’s pin strength was higher than the CM abutments. CM abutments measured a higher torque than the AM abutments.

**Conclusions:**

Additive manufacturing with titanium using SLM provided an alternative fabrication pathway of a novel implant abutment. The AM approach was cost-effective, predictable, efficient and demonstrated pin strength and torque suitable for temporization procedures in implant dentistry.

** Key words:**Abutment, dental implant, temporization, medical device, prototype design, additive manufacturing, 3D printing, titanium.

## Introduction

Implant-supported restorations are a well-recognized esthetic and functional solution for partially edentulous patients (1,2). In the interim phase of treatment, temporary restorations are required to restore gingival health (2,3), while providing esthetic and functional benefits to the patient (1). Despite these advantages, the use of temporaries present a challenging situation (4). With high potential for clinical failure (2,5,6) and a demand for optimal esthetics (7), considerable scientific interest has been focused on refining the components and processes for predicTable implant temporaries (8).

Failure of implant-supported restorations may be directly related to the component design of implant temporaries (9). Cho and colleagues (10) reported that many temporary prostheses applied external stressors that initiated soft-tissue inflammation and inhibited osseointegration. Poorly fitted removable partial dentures can induce undesired forces and resultant stresses in the healing cap and/or implant body (11). These undesired stresses can eventually lead to failure of the implant-supported restorations (12). Therefore, further research focused on temporary abutment designs that minimize these forces is required.

A novel abutment and process for temporization (U.S. Patent No. 12/668832) has been developed (Research Driven, Ontario, Canada) that addresses the functional, aesthetic, and financial requirements of temporary prosthesis and may be considered as an alternative option (13).

**Figure 1:**
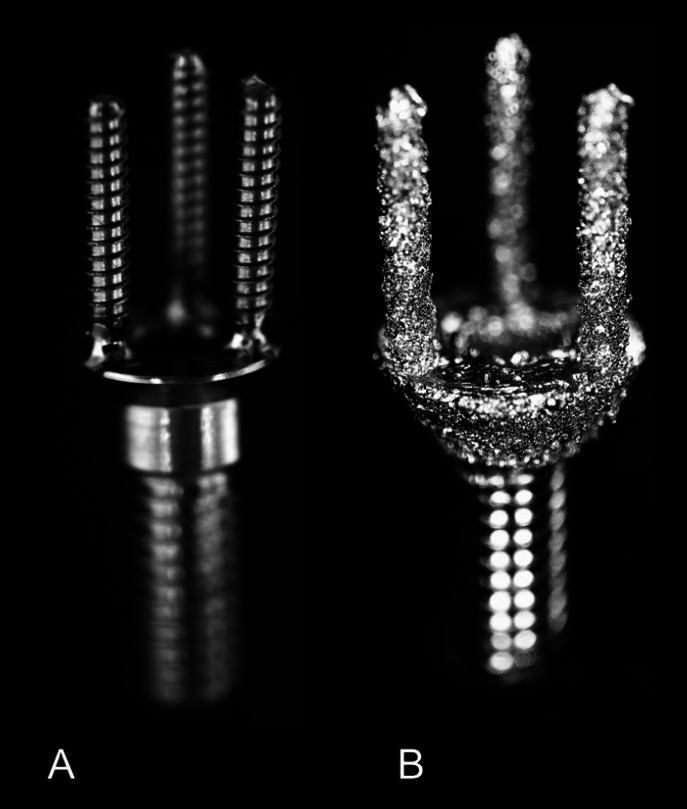
A. Conventionally manufactured novel abutment. B. Additive manufactured novel abutment.

The novel abutment demonstrated advantageous characteristics for its potential use (13), but the production was unpredicTable and costly with traditional machining and laser welding, due to the size and intricate geometric features (Fig. 1A). A workflow was recently developed to apply additive manufacturing using titanium to fabricate the novel abutment (Fig. 1B) (14).

This *in vitro* investigation assessed the torque and pin strength of the additive manufactured (AM) novel abutment against that of a conventionally manufactured (CM) abutment. The investigation would provide important data to determine if the abutment could tolerate (1) tightening within an implant body and (2) the process of temporization.

## Material and Methods

The CM abutments were fabricated by utilizing a 6 mm diameter, 1.5 mm in height, titanium regular neck closure cap (Straumann, Zurich, Switzerland) and 0.60 mm diameter titanium dentine pins (Fairfax Dental, London, England). The pins were manually laser welded to the closure cap with titanium laser wire (Biogenic Technical Institute, Utica, NY) using a magnified laser welder (LaserStar Technologies, Orlando, Florida). The CM abutments were fabricated through a commercial dental laboratory (Expertec Dental Lab, Westland, Michigan).

The AM abutment was developed through a novel workflow (14). The digital design was optimized for additive manufacturing and the abutment was printed in dental-grade titanium (Ti-6Al-4V) (ADEISS, London, Canada). The abutments underwent post-processing, which included final thread tapping to accentuate thread geometries, heat treatment for relieving thermal stresses and strengthening, and bead blasting for a smoother finish.

The CM and HM abutments were evaluated for fit and suitability with a dental implant analogue through visual, tactile and radiographic assessment by an experienced clinician.

-Assessment of *Pi*n Strength 

**Figure 2:**
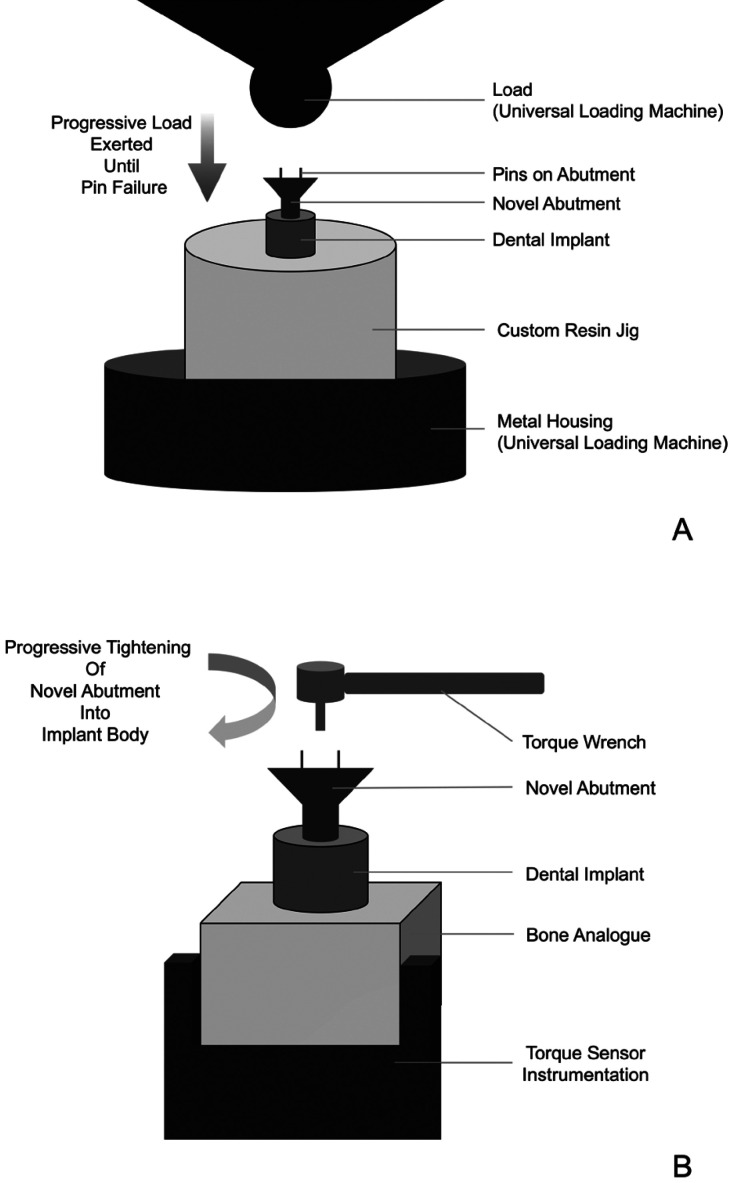
A. Diagrammatic representation for the assessment of pin strength. B. Diagrammatic representation for the assessment of torque.

The novel abutment has a unique process for temporization (13). The abutment has three pin projections that act to retain the temporary crown. Accordingly, the strength of the pins required assessment. The temporization technique is unique, with limited physical data, and this assessment (Fig. 2A) was developed similar to other pin testing protocols.

Five custom jigs were fabricated to facilitate the assessment (Fig. 2A). Jigs were fabricated from light cured resin (Dentsply, York, USA) with an embedded implant analogue (Straumann, Zurich, Switzerland). The resin jigs were fabricated to fit within the metal housing unit of the universal loading machine (Instron, Norwood, MA). Twenty-five [25] CM abutments and twenty-five [25] AM abutments were sequentially placed into the implant analogue and torqued to 25 Ncm. The jig was then inserted into a universal loading machine. An axial load, perpendicular to the abutment platform, was generated at a maximum cross head speed of 0.5 mm/min until mechanical failure of the abutment occurred. The corresponding value was then measured and recorded.

-Assessment of Torque

Assessment of torque was completed with a modified torque measurement protocol (Fig. 2B). Fifty [50] dental implant analogues (Straumann, Zurich, Switzerland) were placed into artificial bone blocks (Sawbones: Pacific Research Laboratories, Vashon, Washington) by an experienced clinician. Twenty-five [25] CM abutments and twenty-five [25] AM abutments were threaded into the implant bodies. The bone blocks were placed into a custom jig. The abutments were then tightened with standard torque wrenches (Straumann, Zurich, Switzerland; AB Dental Devices Ltd., Ashdod, Israel). Torque was measured by a torque sensor (AMTI 6-DOF; Advanced Mechanical Technology Inc., Watertown, MA) secured at the base of the bone block (Fig. 2B). Torque was continuously measured during tightening, by the sensor, and the maximum torque for each abutment was recorded (Instron WaveMatrix Software; Instron, Norwood, MA).

## Results

**Figure 3:**
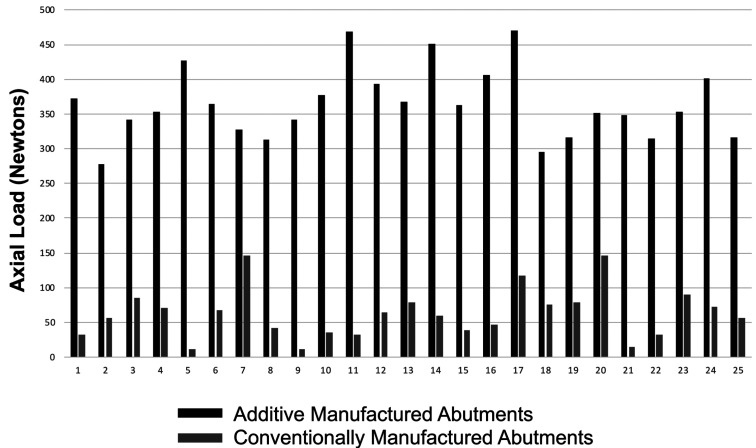
Pin strength of novel abutments.

Figure 3 illustrates the data from the assessment of pin strength of novel abutments. The maximum axial load that the pin projections on the abutment could tolerate (i.e. fracture resistance), before breakage or bending, was recorded. The average pin strength for AM abutments was 364.4 N and for CM abutments was 62.5 N. Minimum and maximum pin strengths for the AM abutments were 278.3 N and 468.9 N, respectively. Minimum and maximum pin strengths for the CM abutments were 12.1 N and 146.9 N, respectively.

**Figure 4:**
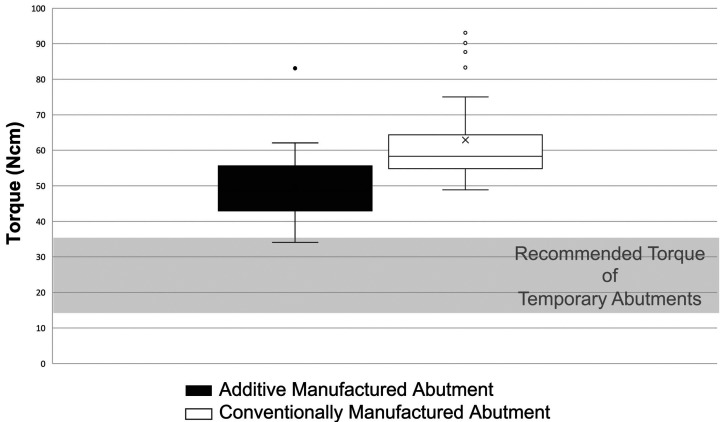
Torque of novel abutments.

Figure 4 illustrates the data from the assessment of torque of novel abutments. The maximum torque of the abutment into the implant body was recorded. The average torque for AM abutments was of 49.9 Ncm and for CM abutments was 62.9 Ncm. Minimum and maximum torque for the AM abutments was 34.1 and 83.1 Ncm, respectively. Minimum and maximum torque for the CM abutments was 48.9 and 93.1 Ncm, respectively.

## Discussion

The strength of the pins for the CM abutments were relatively low and most likely related to the unpredictability of manually welding titanium to titanium. The author had significant difficulty finding a commercial lab that would complete the welding, due to its complexity. The AM abutments had a relatively higher pin strength. This is most likely related to the SLM process and the 30-50 micrometer layer build.

The goal of the pin strength assessment was to estimate if the pins, and the abutment, could tolerate the temporization process. There have been no studies quantifying temporization. The author inferred that the lowest AM pin strength value (278 N) is approximately the force required for the extraction of a single rooted tooth (15). The temporization process would be a fraction of that value, and pin strength would be suiTable for the process. Masticatory forces would not be relevant in the evaluation of the abutment, as the temporary prosthesis would be reduced out of occlusion.

The assessment of torque generated a wide range of values for both the AM and CM abutments (Fig. 4). This may be attributed to the measurement protocol. The implant analogues were placed into bone analogues. As the abutments were tightened, some of the implant bodies rotated within the bone analogue, causing inaccurate measurements.

The CM abutments had relatively higher torques than the AM abutments. This was most likely attributed to a problem with the abutment design, as the torque wrench was slipping within the receptacle of the abutment, causing inaccurate measurements. A design modification is required to incorporate a deeper receptacle or a modified geometric shape to prevent the wrench from slipping when tightening.

The lowest torque for the AM abutment was 34.1 Ncm. Technical information (16) recommends the torqueing of a metal temporary abutment should range from 15 to 35 Ncm. The AM abutments torque values were within this range, except for one value. The data suggests that the AM abutments could be utilized for temporization procedures, as long as they are tightened at the lower limit of the recommendation, at approximately 15-25 Ncm.

The cost to manufacture the CM abutment was approximately $225 CDN/unit. This workflow required several weeks, to acquire the materials and for outsourcing the laser welding. Approximately 50% of the CM abutments could not be used for testing, due to variations in welded pin orientation, rendering many units useless. In contrast, the AM abutments required about one week for fabrication, with a cost of approximately $13 CDN/unit. Remarkably, 100% of the abutments could be utilized for testing.

There were many variables that could have impacted the results. Variations in the position and angulation of the welded pins on the CM abutment, the quality of the laser weld and the angulation of the implant in the custom jig, could have affected pin strength testing. Testing protocols could have impacted the torque assessment, as previously mentioned. Further samples and testing are required to reevaluate torque with a modified abutment design.

Based on this limited investigation, the additive manufactured novel abutment seems suitable as a temporary abutment option. Additive manufacturing with titanium using SLM, provides a predictable, cost-effective, efficient and customizable approach for the fabrication of a novel dental implant abutment.
